# The Great War: III. The Expeditionary Force Base

**Published:** 1918-03-09

**Authors:** 


### THE GREAT WAR.

### III.—The Expeditionary Force Base.

IN the vicinity of all the ports on the northern coast of France, from Calais to the Loire, are situated numerous war hospitals of all sizes and kinds: general hospitals with over 1,000 beds, and stationary hospitals with many hundreds, some devoted to general purposes, others to some special purpose, such as dentistry, the mental and nervous diseases of war, fractures, etc., etc.; in some of the larger hospitals there are special departments, such as $x$-rays, bacteriology, injuries to the head, of the chest, and of the abdomen. There are British, Irish, Scotch, Cânadian, Australian, South African, American, Red Cross, St. John Ambulance Brigade, and officers' hospitals in hotels, casinos, and huts. Attached to these hospitals are consultants, specialists, and scientists, all of world reputation, who concentrate their devoted labours on the repair of the ravages of those who are equally restlessly striving to make them more terrible.

### WOMEN'S MOTOR AMBULANCE CONVOYS.

On arrival at the bases the ambulance trains are met by motor ambulance cars, now largely driven by women, who are gradually releasing men for work at the Front. There are already five of these units at work at five ambulance termini. They are quite a success; the drivers rank as privates, but are distinguished from privates of the W.A.A.C. by blue uniform, and by not being allowed to associate with the rank and file of the army. Those aspiring to a higher position in the motor ambulance world may join another organisation, in which the members hold the position of officers, but receive no pay. Some of the women drivers find the constant "cranking" a great strain on their powers, which sometimes culminates in a new ailment which has been named "motor convoy disease," the prominent symptom of which is paresis of the lower extremities.

### WEAPONS OF WAR AND WOUNDS.

It will be of interest, before we consider the work of the hospitals, to refer to warlike weapons and the injuries they inflict. Wounds are caused by bullets from machine guns or rifles, by shrapnel, by bombs and rifle grenades, and by explosive projectiles. The relative proportions of the various kinds of wounds vary with the character of the fighting. As a rule machine-gun and rifle-bullet wounds predominate. In one of our greatest battles the proportion was as high as 57 per cent., high explosive being 21 per cent., then shrapnel 14 per cent., and, last of all, bombs, 8 per cent. In another battle the proportion of high-explosive and bomb wounds to those caused by bullets was as four to three. This is exceptional. The proportion of slight cases is almost invariably considerably higher than severe ones; it varies from 50 per cent. to over 80 per cent.

The regional distribution of wounds is fairly constant. The limbs are hit more frequently than the trunk and head, the upper and lower limbs in about equal proportions, and the head, chest, and abdomen in the order named. The percentage regional distribution of wounds in one of our great battles was: Upper extremity, 30 per cent.; lower extremity, 28 per cent.; head, 21 per cent.; chest, 16 per cent.; abdomen, 4 per cent., and this is fairly typical.

### THE GREAT HOSPITALS.

The great war hospitals at the bases are super-hospitals manned by super-men and super-women. The strain on the establishment, both *personnel* and material, during the occurrence of heavy casualties at the Front is enormous; at this time they act somewhat in the capacity of clearing stations, for as soon as cases not likely to recover in a reasonable time are fit to travel, they are sent across the Channel in order to make room for possible eventualities. Still, there are always enough remaining in the intervals of big battles to keep the staff fully occupied.

### WAR HOSPITALS ARE HIGHLY SPECIALISED.

The organisation of the technical work is highly specialised; almost every ward is devoted to some special injury or to some special part of the body—a ward for heads, another for chests, others for fractures or for the treatment of wounds by special means, each one presided over by an eminent enthusiast. Occasionally some of these enthusiasts attain such success in their particular methods that they are moved from their parent hospital to one entirely their own; such, for instance, as that devoted to the marvellous apparatus for the treatment of fractures by two surgeons who had worked independently on somewhat similar lines. In it you see patients with their fractures accurately adjusted and fixed in frames of aluminium, suspended in the air by a system of pulleys in such a manner that they can move their bodies without disturbance of injured limbs, the open wounds of which are at all times freely accessible. The extension apparatus is also a wonder, by a system of cogs and levers the minutest adjustment, checked by $x$-ray screen examination, being made by the mere click of a cog. The changing of dressings in a case of compound fracture of a limb treated in the ordinary way, in spite of the gentlest manipulation by the gentlest of nursing sisters, causes considerable local and general disturbance and a rise of temperature, which is avoided by the system we have described.

### MICROBES.

In these hospitals also can be studied the great war of scientists against the deadly attack of surgical germs, such as the tetanus, the streptococcus and the *Bacilli bellonensis* and *perfringens*, the instigators of gas gangrene. The tetanus microbe has been overcome by the injection of anti-tetanic serum, which every wounded officer and man receives

Previous articles appeared on February 9, page 391, and 23. page 437.

**THE EXPEDITIONARY FORCE BASE—(*continued*).**

at the advanced dressing station at the earliest possible moment after being wounded. The streptococcus is being fought independently by two different schools; one which encourages the body elements to resist the attack by natural means, such as phagocytosis and the action of anti-bodies, and the other by the use of antiseptics. In one hospital we saw grouped wounds of the chest and extensive injuries of the limbs, under treatment by solutions of chloride of sodium of strengths varying from 2.5 per cent. to 5 per cent., a constant flow at the desired rate being maintained by a system of tubes connected with an automatic irrigation apparatus. Chloride of sodium acts by causing an osmotic flow of lymphatic fluid and cells from the tissues. In the bacteriological department we were shown slides under the microscope illustrating the invasion of the wound surface by phagocytes, which could be seen in the act of digesting germs. The strength of the solution requires careful regulation; the use of a strong solution on an extensive surface is apt to cause dangerous collapse, due to the withdrawal of serum from the body. In another ward the surgeons were interested in the B.I.P. (bismuth, iodoform, paraffin) treatment of the wounds of joints. It has the advantage of requiring no attention during the journey from the clearing station, but its use is not very popular owing to the danger of systemic poisoning, due to absorption. In another hospital, wards were specially fitted with overhead rails for travelling receivers carrying solution required for the Carrel-Dakin treatment of wounds by progressive sterilisation, the success of which depends upon the quality of the hypochlorous solution, and attention to details. The bactericidal activity of hypochlorous acid is evanescent, and the treatment dependent on constant renewal of the fluid, which is attained by an automatic flusher. The neutralisation of this antiseptic by septic material was illustrated to us *in vitro*, in which streptococcal growth was to be seen invading the treated nutrient medium.

One of the disadvantages of this antiseptic is its disintegrating action on blood clot and consequent liability to cause secondary hæmorrhage. Flavine, the antiseptic which was to have revolutionised the treatment of wounds, has not realised the expectations based on experiments on animals; its continued use for more than a few days has a devitalising action on the tissues which delays healing. Wounds which can be thoroughly cleansed, however, and for which one application is sufficient, can be closed primarily, and frequently heal by first intention.

### Gas Gangrene.

The Jules Verneses, the Sherlock Holmeses, the Nelsons, and Napoleons of science and surgery are still baffled by the vicious and despicable anaerobes of fæcal origin which are responsible for the most pitiable of misfortunes to which war wounds are liable. Scientists are striving to overcome the toxæmia, to raise the resistance of the tissues to the tryptic action, and to overcome the acidosis, which are some of the weapons of these vile germs, by the inoculation of vaccines and salts. Some day, if the war lasts sufficiently long, they will meet with complete success, but in the meantime our hopes must continue to rely upon those surgical measures, both preventive and curative, which experience has proved to have been most suitable. Extensive shell and bomb wounds are most frequently affected by gas gangrene, especially those of the fleshy parts of the lower extremities. Many kinds of germs form gas in the tissues; the commonest are the *Bacilli perfringens, bellonensis*, and *œdematiens*. They are carried in from the infected clothing or skin, and multiply in the dead and stagnant tissues.

### The Surgical Treatment of Gas Gangrene.

Gas gangrene does not occur in wounds which are operated upon early. The removal of foreign bodies, including dead tissue, and the provision of thorough drainage is then preventive. These measures are essential. The particular lotion used afterwards is immaterial as long as free drainage is maintained and the wound is not allowed to dry; sterile water is as efficacious as salt solution or antiseptics. When the formation of gas in the tissues has actually commenced the progress is very rapid, and the danger limit is often reached in a few hours, a situation demanding bold and immediate incisions. At a large French hospital devoted to serious wounds, which we visited, great success was claimed for oxygen gas, which was forced into the affected tissues until the limb resembled a balloon. We noticed, however, that the operative treatment was very drastic and thorough; indeed, to such an extent as to raise doubts in our minds of the probability of effectual contact between the oxygen and the anaerobes in the tissues.

### Dental Surgery.

The war has given a wonderful impetus to dental surgery in the Army. In the early days dental surgeons were rare and poorly equipped, even at the bases, but there is now a well-organised system of dental establishments from base to front, and there are mobile dental units, evidently in emulation of our Dominion Forces, who arrived with them as integral parts of their field army. From an economic point of view the filling or extraction of teeth and the upkeep of plates is of greater moment to the fighting forces than the reparation or renewal of jaw-bones. Many thousands of teeth have been filled or extracted, and we actually came across a soldier who had been provided with a full set of dentures at the lightning speed of ten hours. But for scientific interest and moral effect, the plastic operations on wounded jaws, which we studied at the dental hospital presided over by a well-known French surgeon attached to the British Red Cross, are excelled by no other marvels of the war. The various stages in the reconstruction of the jaws and features of those we saw under treatment were illustrated by series of photographs, casts, and models, including the replacement of bone by aluminium, and of noses, cheeks, and lips by transplantations from foreheads and arms.

**THE EXPEDITIONARY FORCE BASE—(*continued*).**

America at Work.

American doctors at the Front are a success; the expectations of the optimists have been more than realised. There are many hundreds of them permanently attached to our medical units in the field, on the lines of communication, and at the base, while a continuous stream of learners passes from unit to unit in pairs, undergoing systematic courses of instruction. Everywhere we heard appreciation of their professional attainments, their nobility of character, their versatility and sense of humour.

They were naturally more interested in professional subjects than in administration, but when in due course they are recalled to their own army they will occupy positions of responsibility, and then more fully realise its essential importance. Among them, good operating surgeons, anæsthetists, and research scholars abound. To one of the latter is due the discovery that the bloods of certain groups of individuals are complementary, and that in the operation of transfusion if this grouping is ignored coagulation occurs, with the evident consequences. There are four complementary groups, the percentage of each being approximately: No. 1, 10 per cent.; No. 2, 40 per cent.; No. 3, 7 per cent.; No. 4, 43 per cent.; with the strange exception that Group 2 is complementary to all four groups. Others are working at traumatic shock and inventing new "corpse revivers," as the latest and most successful of the alkaline intravenous injections is called. Shell-shock and mental conditions are also popular subjects with them, and we met one who was interesting himself in psychic phenomena. As an illustration of their sense of humour we reproduce the following lines, reminiscent of Omar Khayyám, posted by one of them on the humid walls of a dank and dismal cellar not far from the firing line:—

Harken all ye whom duty calls
To spend some time within these friendly walls.
Others will sojourn here when you have passed,
You are not first nor will you be the last.
Therefore take heed and do whate'er you may
For safety and for comfort while you stay;
Just put a sandbag here, a picture there,
To make a room more safe, a wall less bare.
Think as you tread the thorny path of duty,
Of comfort, of security, and beauty,
So your successors when they come may say,
"A splendid unit we relieved to-day."

